# Application of systemic treatment in conversion therapy options for liver cancer

**DOI:** 10.3389/fonc.2022.966821

**Published:** 2022-10-06

**Authors:** Haoyang Bei, Weiheng Mai, Weifeng Chen, Mingyi Li, Yongguang Yang

**Affiliations:** Department of Hepatobiliary Surgery, The Affiliated Hospital of Guangdong Medical University, Zhanjiang, China

**Keywords:** primary liver cancer, systemic treatment, conversion therapy, downstaging, surgical resection

## Abstract

Radical hepatectomy is the main treatment method to improve the prognosis of patients with intermediate and early-stage liver cancer. Most liver cancer patients in China are in the advanced stage at the initial diagnosis, losing the opportunity for surgical treatment. Therefore, it is essential to down-stage unresectable liver cancer to resectable liver cancer clinically, which is an important way to improve patients’ survival and a hotspot of current clinical research. In recent years, with the increase in effective treatment methods for liver cancer, the resection rate of conversion surgery for unresectable advanced liver cancer has been significantly improved, and a growing number of patients benefit from conversion therapy. This article mainly reviews the connotation of conversion therapy for liver cancer, the patient selection, the selection of conversion strategy, the timing of sequential operations, the scheme and safety, etc.

## Introduction

As one of the most common malignant tumors in the world, primary liver cancer ranks sixth in the incidence of malignant tumors worldwide in 2018, and is the fourth leading cause of tumor death (1). Primary liver cancer is the fourth most common malignant tumor and the second leading cause of tumor death in China, which seriously threatens the lives and health of the Chinese people. Hepatocellular carcinoma (HCC) (hereafter referred to as liver cancer) accounts for 75% of 85% of primary liver cancer cases. Risk factors for liver cancer include chronic viral hepatitis (hepatitis B virus infection, hepatitis C virus infection), alcoholic liver disease, consumption of food contaminated by aflatoxin, obesity and diabetes, etc. Among them, chronic hepatitis B virus (HBV) infection is the main risk factor for HCC in China (2). For patients with early-stage liver cancer, the main treatment methods include surgical resection, local ablation and liver transplantation. However, due to the latent onset and rapid progress of liver cancer, most patients are diagnosed at the intermediate and advanced stages including Barcelona Clinic Liver Cancer(BCLC) stage B, C or China liver cancer staging (CNCL) stage IIIa, IIIb and some stage IIb, when the surgical effect is poor or the opportunity for surgery is lost, and the median survival time is only 1 year (3). For such patients, the most important thing is to transform unresectable liver cancer into resectable liver cancer and perform successful surgery, which is also the key to long-term survival. In recent years, the development of tyrosine kinase inhibitors (TKIs) and immune checkpoint inhibitors (ICIs) has brought opportunities for the treatment of liver cancer at intermediate and advanced stages, and conversion therapy has become one of the current research hotspots.

As a treatment method for unresectable liver cancer, conversion therapy for liver cancer mainly adopts systematic drug therapy and or non-surgical local therapy to inhibit tumor progression, reduce tumor burden and improve clinical tumor staging, thereby providing patients the opportunity to undergo radical surgery (4, 5). The other category of conversion therapy is neoadjuvant therapy, which refers to HCC patients with technically resectable tumors and a high risk of recurrence. It aims to shrink the tumor, improve the radical resection rate, and reduce recurrence. When the treatment is applied to patients with surgically resectable but oncologically unresectable HCC, both treatments may be overlapped in the target population (5). At present, the commonly used conversion therapy methods in clinic include targeted therapy, immunotherapy, local therapy, radiotherapy and other combination therapy methods. With the in-depth investigation of various clinical studies, more and more patients with liver cancer at intermediate and advanced stages have benefited from conversion therapy. The regimen and efficacy of conversion therapy are described as follows:

## Application of drug therapy in conversion therapy

### Effect of drug therapy

#### Targeted therapy

Mainly TKIs drugs with representatives including sorafenib, lenvatinib, apatinib, etc. Sorafenib can directly inhibit tumor cell proliferation by inhibiting the rat sarcoma virus (Ras)/rapidly accelerated fibrosarcoma (Raf)/mitogen-activated protein kinase kinase (MEK)/extracellular signal-regulated kinase (ERK) signaling pathway, which was used in the treatment of renal cell carcinoma at first. It was approved by the U.S. Food and Drug Administration (FDA) in 2007 for the treatment of advanced HCC. In a large randomized controlled international multicenter clinical trial (SHARP study), 602 patients with advanced liver cancer who had not received systemic treatment were included and randomized to receive sorafenib or placebo. The results showed that the median overall survival (mOS) in the sorafenib group (n1 = 299) and the placebo group (n2 = 303) was 10.7 months vs. 7.9 months (P<0.001), and the median time to progression (mTTP) was 5.5 months vs. 2.8 months (P<0.001), indicating that sorafenib can postpone the progression of advanced liver cancer and prolong the survival of patients (6). Another clinical study on sorafenib (Oriental study) also came to similar conclusions (7). Sorafenib, as the first molecular targeted drug for the treatment of advanced liver cancer, has made a certain contribution to prolonging the survival of patients. However, due to its low objective response rate (ORR) (about 2.3%), significant adverse reactions, no obvious improvement in the overall survival rate in hepatitis B virus-positive patients, it still cannot fulfill the current needs for treatment of advanced liver cancer.

Lenvatinib is an oral multi-receptor tyrosine kinase inhibitor developed by Eisai. Its main targets are: vascular endothelial growth factor receptor (VEGFR) 1-3, fibroblast growth factor receptor (FGFR) 1-4, platelet-derived growth factor receptor (PDGFR) α, c-Kit, RET etc. A phase III randomized controlled global multicenter non-inferiority clinical study, the REFLECT trial (8), compared lenvatinib with sorafenib. For the primary endpoint of the trial, the mOS in the lenvatinib group was non-inferior to the sorafenib group with a trend of prolongation (13.6 months vs. 12.3 months, P>0.001); in terms of secondary endpoints comparing the lenvatinib with sorafenib, the median progression-free survival (mPFS) (7.4 vs. 3.7 months), mTTP (8.9 vs. 3.7 months), and ORR (24% vs. 9%) all improved. In terms of safety, there was no significant difference between lenvatinib and sorafenib with the incidences of treatment-related adverse events similar between the two groups. Meanwhile, for HBV-related HCC, lenvatinib showed more advantages in prolonging the survival. These data indicated that lenvatinib was not inferior to sorafenib in the efficacy for advanced HCC patients, was superior to sorafenib in secondary endpoints such as ORR and mPFS, and was applicable to a wider population. Therefore, it is recommended by many first-line guidelines to be used in the first-line treatment of unresectable HCC. Tomoko’s team (9) also conducted a clinical study of lenvatinib treatment after failure of PD-1/PD-L1 treatment for liver cancer, finding that the mPFS after lenvatinib treatment was 10 months, mOS was 15.8 months, ORR reached 55.6%, and Disease control rate (DCR) reached 86.1%. It showed that the use of lenvatinib could still increase the chance of conversion and prolong the survival after failure of immunotherapy.

Other drugs, for example, apatinib, a new small-molecule targeted drug independently developed by Jiangsu Hengrui Pharmaceuticals, was initially used for the treatment of advanced gastric cancer, and is now also used in patients with advanced liver cancer who fail or are intolerable to first-line systemic anti-tumor treatment, as a second-line therapeutic regimen for advanced liver cancer. The results of a phase III clinical study of advanced liver cancer in China showed that apatinib, compared with placebo, significantly prolonged the median survival in patients with advanced liver cancer receiving second-line treatment or above, and the ORR reached 10.7%. The risk of death was reduced by 21.5%, and the risk of disease progression was reduced by 52.9% (10).

#### Targeted drugs combined with immunotherapy

Currently, targeted drugs combined with immunotherapy has become the first-line treatment strategy for advanced HCC. In the IMbrave150 study, atezolizumab combined with bevacizumab (T+A) achieved better positive results compared with sorafenib, showing significantly improved mOS (19.2 months vs. 13.4 months p < 0.001) and mPFS (6.9 months vs. 4.3 months p < 0.001) in liver cancer patients after treated with the T+A regimen (11). In addition to the T+A regimen, the combination of TKI drugs with PD-1/PD-L1 has also achieved good results. In the phase Ib study Keynote524 (12), the mPFS of lenvatinib combined with pembrolizumab was 9.7 months, the mOS was 20.4 months, and the ORR was 46.3%. In 2019, this regimen was used as the first-line treatment regimen for liver cancer at advanced stage. At the 2020 ASCO-GI meeting, a phase Ib study of lenvatinib combined with nivolumab as the first-line treatment for patients with unresectable liver cancer (13) was reported with the results showing the overall ORR 76.7%, DCR 96.7%, and the clinical benefit rate 83.3%. In the study reported by Zhongshan Hospital affiliated to Fudan University using lenvatinib combined with PD-1 monoclonal antibodies (including nivolumab, camrelizumab, pembrolizumab, sintilimab and toripalimab) in the treatment of advanced HCC (14), the results showed that 6 patients (10.2%) underwent the surgical resection as the tumor had shrunk. Another clinical study of lenvatinib combined with pembrolizumab and apatinib combined with toripalimab in the treatment of unresectable liver cancer was published (15), wherein 10 patients (15.9%) underwent surgical resection 3.2 months (2.4-8.3 months) after the start of treatment and 6 patients (60%) achieved pathological complete response (pCR). In a prospective, uncontrolled, open-label study led by Professor Lu Shichun (16), PD-1 monoclonal antibodies combined with lenvatinib were investigated for the efficacy in the treatment of liver cancer with macrovascular invasion, and the results showed that the ORR was 53.1%(26/49), and the imaging-based conversion rate reached 51.0%, and 15 patients (30.6%) underwent R0 surgical resection. In a retrospective analysis of lenvatinib combined with camrelizumab versus lenvatinib alone, the efficacy in the combination group was improved compared with the single agent group, showing mPFS increasing from 7.5 months to 10.3 months (P<0.05), ORR increasing from 20.5% to 41.7% (P<0.05) (17). It showed that TKI drugs combined with PD-1 is a more effective conversion regimen.

#### Targeted therapy combined with local therapy

As the most commonly used local treatment method and one of the most common non-surgical treatment methods for liver cancer, Transarterial Chemoembolization (TACE) has certain effects in reducing tumor burden and prolonging patient survival, which is also recommended by many guidelines as the standard treatment for intermediate-stage liver cancer (18–20). However, when it is used alone, the conversion efficiency of TACE is low and recurrence often happens, moreover, multiple TACE can lead to poor efficacy or even resistance (21). Studies have shown that TACE combined with targeted therapy can improve the efficacy of TACE. A retrospective study led by Professor Shi Ming compared the efficacy of TACE combined with sorafenib versus sorafenib monotherapy in the treatment of advanced liver cancer complicated with hepatic vein tumor thrombus. The results showed that the OS and TTP in the TACE combined with sorafenib group were superior to sorafenib monotherapy (22). Ding et al. (23) conducted a study comparing the efficacy of sorafenib combined with TACE (TACE-S) and lenvatinib combined with TACE (TACE-L) in the treatment of advanced liver cancer complicated with portal vein tumor thrombus (PVTT). The results showed that TACE-L was superior to TACE-S in both mOS (14.5 months vs. 10.8 months) and mTTP (4.7 months vs. 3.1 months), and 17 patients (53.1%) in the TACE-L group achieved partial response, compared to 12 (25.0%) in the TACE-S group. Another study has also found that hepatic arterial infusion chemotherapy (HAIC) based on FOLFOX regimen is superior to TACE in efficacy, and HAIC combined with targeted therapy has also achieved good results. A prospective study found that sorafenib combined with HAIC significantly improved the survival and conversion rate compared with sorafenib alone, increasing mOS from 7.13 months to 13.37 months (p<0.05), PFS from 2.6 months to 7.03 months (p<0.05), and improving ORR from 5.7% to 54.4% (24). In a retrospective study reported by Mai at the American Society of Clinical Oncology in 2020 (25), 24 patients with advanced liver cancer who received FOLFOX regimen based HAIC combined with lenvatinib were analyzed showing that ORR and DCR were 66.7% and 79.2%, respectively. Among the targeted treatment combined with local treatment regimens, lenvatinib combined with HAIC achieved the best conversion effect.

#### Targeted therapy, immunotherapy combined with local therapy

Recent studies have shown that targeted immunotherapy combined with local therapy (TACE or HAIC) can further improve the surgical conversion rate of advanced unresectable liver cancer. In a study conducted to explore the efficacy of lenvatinib combined with TACE and pembrolizumab versus lenvatinib combined with TACE in the treatment of unresectable liver cancer, Chen et al. (26) found that the OS and PFS of triple therapy were 18.1 months and 9.2 months respectively, superior to 14.1 months and 5.5 months of dual therapy, and 18 patients (25.7%) in the triple therapy group were successfully downstaged to undergo surgery, while 8 (11.1%) in the dual therapy group underwent surgery. Another retrospective analysis investigating the conversion of triple therapy using anti-angiogenic drugs combined with PD-1 and HAIC for unresectable liver cancer showed that the objective response rate was 96% (24/25) with 14 patients (56%) undergoing surgical resection, including 7 cases achieving pathologic complete response (27). In the retrospective analysis to investigate the efficacy of TACE combined with lenvatinib and sintilimab, the mOS of this regimen was 23.6 months, the mPFS was 13.3 months, and the ORR was 46.7% (28). In the LTHAIC study, a prospective, single-arm phase II clinical study (29), the treatment regimen of lenvatinib + toripalimab + HAIC showed to have an ORR of 66.7% (95% CI, 43.3-75.1), including 5 (13.9%) patients achieving complete radiographic response and 8 patients successfully downstaged to meet the criteria for surgical resection (**Table 1**). At present, the triple therapy shows the highest conversion efficiency.

**Table 1:** Clinical studies of conversion therapy in unresectable HCC patients.

Medicine	Case	mOS(month)	mPFS(month)	ORR%	DCR%	CR(%)	Surg(%)	TRAE %	Outcome
TKIs									
Sor (SHARP) [6]	299	10.7	5.5^e^	2.0^a^	43^a^	–	–	80	sorafenib improves overall survival by nearly 3 months.
Sor (Oriental) [7]	150	6.5	2.8^e^	3.3^a^	53^a^	–	–	98.0^c^	Sorafenib prolongs OS, TTP and improves DCR.
LEN (REFLECT) (8)	478	13.6	7.4	24.1^b^	75.5^b^	–	–	94	lenvatinib is non-inferiority of sorafenib in OS, and improves PFS, TTP and ORR.
Apatinib (AHELP) (10)	267	8.7	4.5	11.0^a^	61^a^	–	–	97	Apatinib significantly improves OS in patients with pretreated HCC.
TKIs+ICIs									
T+A (IMbrave150) (11)	336	19.2	4.3	30.0^a^	74^a^	25(7.4)^a^	–	86	T+A maintained clinically survival benefits over sorafenib.
LEN+Pembrolizumab (12)	104	22.0	9.3	46^b^	88^b^	5(4.8) ^b^	–	99	LEN plus pembrolizumab improves antitumor activity in uHCC.
LEN +NIV (13)	30	–	–	76.7^b^	96.7^b^	4(13.3)^b^	9(30.0)	100	LEN + NIV has encouraging anti-tumor activity in uHCC.
LEN +PD-1 (14)	59	–	–	55.9^b^	76.2 ^b^	9(15.3)^b^	10(16.9)	–	LEN+PD-1 is effective and may convert unresectable HCC into resectable.
TKIs+PD-1 (15)	63	–	–	–	–	–	–	–	TKI+PD-1 is a feasible to convert unresectable HCC into resectable.
LEN +PD-1 (16)	46	NR	NR	53.1	69.4	5(10.8)	–	–	LEN+PD-1 could benefit unresectable HCC patients to achieve curative surgery.
LEN +Cam (17)	48	NR	10.3	41.7^b^	75.0^b^	4(8.3)^b^	–	–	LEN+Cam might benefit patients with unresectable HCC more than lenvatinib monotherapy
TKIs+local therapy									
Sor+TACE (22)	20	14.9	4.9^e^	50^b^	80^b^		–	–	Sor+TACE is effective in treating advanced HCC and HVTT
LEN+TACE (23)	32	14.5	4.7^e^	53.1^b^	90.6^b^		–	100	LEN+TACE is more effective than Sor+TACE in advanced HCC with PVTT
Sor+HAIC (24)	125	13.37	7.03	40.8^b^	75.2^b^	10(8.0)^b^	16(10.0)	95.16	Sor+HAIC improves OS in patients with HCC and portal vein invasion
LEN+HAIC (25)	24	–	8.1	66.7^b^	79.2^b^	–	–	–	6-, 9-, and 12-months OS rates were 91.7, 83.3%, and 75%, respectively.
TKIs+ICIs+local therapy									
LEN+TACE+Pembrolizumab (26)	70	18.1	9.2	47.1^b^	70.0^b^	7(10.0)^b^	18(25.7)	–	Pembrolizumab+LEN+ TACE contribute to a higher rate of conversion therapy and longer survival time than the lenvatinibTACE regimen
TIKs+PD-1+HAIC (27)	25	–	–	96^b^	100^b^	12(48.0) ^b^	16(64.0)	92	TIKs+PD-1+HAIC showed significant therapeutic effect with an extremely high surgical conversion rate.
LEN+TACE+sintilimab (28)	60	23.6	13.3	46.7^b^	85.0^b^	6(10)^b^	–	84.6^d^	LEN+TACE+sintilimab is a promising therapeutic regimen in unresectable HCC
LEN+toripalimab+HAIC (29)	36	NR	10.5	66.7^b^	–	5(13.9)^b^	8(22.2)	–	LEN+toripalimab+HAIC shows promising antitumor activity

TKIs, tyrosine kinase inhibitors; ICIs, immune checkpoint inhibitors; Sor, sorafenib; LEN, lenvatinib; PD-1, programmed cell death 1; TACE, Transarterial Chemoembolization; HAIC, hepatic arterial infusion chemotherapy. pCR, pathological complete response. NR, not reached; pts: Patients; TRAE: treatment-related adverse events; TTP, time to progression; mOS, median overall survival; mPFS, median progression-free survival; ORR, objective response rate; DCR, Disease control rate; T+A, atezolizumab plus bevacizumab; NIV, nivolumab; Cam, camrelizumab; HVTT, hepatic vein tumor thrombus; PVTT, portal vein tumor thrombus; HCC; ^a^According to RECIST; ^b^According to mRECIST; ^c^treatment-emergent adverse events (TEAE); ^d^AEs, adverse events; ^e^TTP, time to progress.

In addition, as portal vein metastasis is prone to occur for liver cancer, many patients complicated with portal vein tumor thrombus cannot undergo surgical resection or the resection effect is poor. Some studies have found that when liver cancer is complicated with portal vein tumor thrombus, combination with radiotherapy can make the tumor thrombus shrink or even disappear, creating conditions for surgery and improving patients’ survival. A large randomized controlled trial (RCT) comparing the efficacy of neoadjuvant radiotherapy followed by resection with direct resection in liver cancer patients with portal vein tumor thrombus showed that the 1-year survival rates of the two groups were 75.2% vs. 43.1%, and the 1-year tumor-free survival rates were 33.0% vs. 14.9%, respectively (30). Toshiya et al. compared the efficacy of radiotherapy followed by surgery with direct surgery. The pathological results after surgery showed that 83.3% of patients in the radiotherapy followed by surgery group achieved pathologically complete necrosis of the main portal vein tumor thrombus with the 5-year survival rate of 34.8%, compared to only 13.1% in the surgery alone group (31). There are also clinical data confirming the efficacy of Transarterial Radioembolization (TARE) in shrinking tumors and its role in the conversion therapy for liver cancer. For cases complicated with portal vein tumor thrombus, TARE shows higher local dose and more precise location than external beam radiotherapy, and also reduces radiation damage to normal liver tissue, with less effect on reserve function (32). Therefore, combination with radiotherapy can further improve the conversion rate and prolong the survival in patients withadvanced liver cancer complicated with portal vein tumor thrombus.

Decades ago, for patients with significant tumor load, many lesions, vascular invasion, or distant metastases, the only therapeutic options were TACE, sorafenib, or symptomatic therapy, and the prognosis was dismal, with a median overall survival rate of 6.5-10.7 months (6, 7, 33). Nowadays, In the era of systematic treatment, the application of TKI drugs and PD-1/PD-L1 has enriched the treatment of liver cancer, expanded the beneficiary group of patients, and median OS has reached 18.1 months or even longer (13, 14, 24, 26). Due to the decrease in tumor volume and stage(reducing the volume and number of primary lesions and eliminating portal vein tumor thrombus and metastatic lesions), part of advanced HCC patients have obtained the opportunity for radical surgery (**Table 2**) (34).

**Table 2:** Summary of the advantages and disadvantages of monotherapy and combination therapy.

	Advantage	Disadvantage
Monotherapy (6–8, 10)	Compared with a placebo, a monotherapy regimen prolongs the survival of patients with advanced liver cancer, with lenvatinib being the most effective.	Adverse reactions to monotherapy are between 80% to 98%, and every monotherapy regimen has a similar incidence of severe adverse reactions, which is manageable.
Bigeminy therapy		
TKIs+ICIs (11–17)	TKIs combined with ICIs are more effective in conversion therapy than monotherapy. In addition, some patients were successfully downstaged, underwent surgical resection, and achieved a complete pathological response (pCR).	There are no statistically significant differences between targeted immunotherapy and monotherapy in the incidence of most adverse events. In general, toxicities are manageable, with no unexpected safety signals.
TKIs+local therapy (22–25)	Compared with monotherapy, TKIs combined with local therapy had better conversion effectiveness and improved OS and PFS in patients with HCC and portal vein invasion.	Some grade 3 to 4 adverse events are more frequent in TKIs combined with local therapy groups than in the monotherapy group. The overall incidence of adverse events is similar and well tolerated.
Triple therapy (26–29)	Triple therapy shows promising antitumor activity and contributes to a higher conversion rate than Bigeminy therapy for patients in advanced HCC and PVTT.	There were no significant differences in majority grade ≥ 3 AEs between triple therapy and bigeminy therapy, and toxic side effects were manageable.

TKIs, tyrosine kinase inhibitors; ICIs, immune checkpoint inhibitors; OS, overall survival; PFS, progression-free survival; AEs, adverse events; PVTT, portal vein tumor thrombus.

#### Conversion that increases liver volume

Liver failure caused by insufficient residual liver volume after surgery has become a major restraining factor affecting the surgical resection for liver cancer. For patients undergoing surgery after conversion, the residual liver volume should be maintained over 40% as far as possible. When the requirements cannot be met, portal vein thrombosis (PVE) and associating liver partition and portal vein ligation for staged hepatectomy (ALPPS) can be considered. The complications of PVE were mild, but it took 4-6 weeks to wait for the growth of liver. For some patients who may lose the opportunity for surgery due to tumor progression or insufficient growth of liver, combination with TACE therapy may be considered (35, 36). ALPPS can induce a 47%-192% increase in liver volume within 1-2 weeks, which is much higher than PVE, and the tumor resection rate can also reach 95-100% (37), but it has high incidence of perioperative complications. Therefore, it is necessary to comprehensively evaluate the patient’s condition before surgery, such as level of liver cirrhosis, patient age, the capacity to withstand two surgeries in a short period of time, and the rapid tumor progression (38).

### Management of adverse reactions

While conversion therapy has achieved promising efficacy results, we need to pay attention to the adverse reactions during the treatment. Common adverse reactions of TKI drugs include hypertension, palmar-plantar erythrodysesthesia syndrome (PPES), loss of appetite, nausea, vomiting and fatigue (39). Immune-related adverse events (irAEs) caused by immune checkpoint inhibitors (ICIs) involve almost all organs, and common adverse reactions include rash and itching, diarrhea and colitis, hepatotoxicity, pneumonia, and thyroiditis (40, 41). The adverse reactions of TACE and HAIC are similar with post-embolization syndrome the most common, mainly manifested as fever, hepatalgia, nausea and vomiting, etc (42), and some adverse reactions caused by chemotherapeutic drugs. The assessment of the above adverse events (AEs) should be performed according to the Common Terminology Criteria for Adverse Events Version 5.0 (CTCEA Version 5.0). For mild adverse reactions, symptomatic treatment can be given. For severe adverse reactions, it is necessary to fully evaluate the patient’s condition, discontinue the current treatment and perform active symptomatic treatment, adjust the treatment dose or even change the regimen, etc. (40–43) Most cases are transient or can be resolved through dose reduction or symptomatic treatment. It has been reported in literature that the occurrence of some TKI-related AEs indicated favorable prognosis (44). Therefore, during the conversion therapy, the tolerance to some adverse reactions can be enhanced for patients, and at the same time, serious adverse reactions that occur during the treatment should be alerted for early detection and timely intervention to ensure the efficacy and safety of conversion.

### Patient assessment and regimen selection

Radical operation is the essential means of treating primary liver cancer, which is also a necessary means of achieving long-term survival. It is also the core of conversion therapy, which transforms unresectable liver cancer into resectable liver cancer for surgical resection. Usually, there are two reasons for unresectability: surgically unresectable and oncologically unresectable. The former is widely accepted, including the patient’s inability to withstand surgical trauma regarding their general condition, liver function, and insufficient remaining liver volume (surgically unresectable). The latter means Technically resectable but cannot acquire better effectiveness after resection than non-surgical treatment, which is dynamic and controversial (5). Clinically, for unresectable liver cancer, TKIs combined with ICIs can be used initially for conversion attempt, and the reasons for unresectable tumor should be analyzed and evaluated. In case of excessive tumor burden, TACE or HAIC can be added for tumor shrinkage (45, 46); in case of complication with portal vein tumor thrombus, HAIC or TARE can be added (47, 48), or external beam/Stereotactic Body Radiation Therapy (SBRT) radiotherapy can be combined (49) to achieve tumor thrombus shrinkage or even complete disappearance; in case of complication with extrahepatic oligometastasis, radical resection of the primary tumor + resection or ablation of metastasis can be selected if tolerable after sufficient assessment (50, 51).

Since conversion therapy methods can affect tumor, liver, and other organ functions, patients who have the opportunity for surgery after conversion therapy must be evaluated for organ function, target tumor burden, high-risk factor conversion, residual liver volume, and liver function (52). The evaluation includes regular review of enhanced CT, MRI and other imaging data to dynamically compare the changes of lesions and intra- and extra-hepatic metastasis; completion of the Child-Pugh (CTP) grading, indocyanine green (ICG) clearance test, and model for end-stage liver disease (MELD) score, HBV DNA level, etc. to assess liver function and tolerance to surgery; making full use of 3D visualization technology to use a wide incisal margin with tumor boundary >1 cm as the resection range as far as possible, and ensure that FLR accounts for more than 40% of the standard liver volume so as to ensure the safe implementation of surgery (3).

The treatment regimen should not be selected solely based on the staging of liver cancer as some patients with BCLC stage A (or some CNLC stage Ib), who are not suitable for surgical treatment due to excessive tumor burden at the initial diagnosis, should receive conversion therapy before radical resection; some patients with BCLC stage B/C (CNLC stage IIb/IIIa/IIIb) should not be completely considered as equivalent to the advanced stage for systemic therapy alone, but can undergo radical surgery after conversion therapy. It is recommended to use the multidisciplinary team (MDT) model (5) to fully assess the condition and formulate individualized follow-up and treatment strategies. Combined with the current research results, when the liver function, performance status, general condition is favorable, and the patient can tolerate the treatment, try to choose a regimen combining multiple treatment methods, such as targeted treatment combined with local therapy, to improve the tumor response rate and surgical conversion rate.

## Others

### Surgical resection after conversion

Surgical resection is an important way for patients to obtain long-term survival after successful conversion. An important condition for conversion resection is to achieve tumor response, or at least to keep the lesions stable for a period of time (3~4 months) (42). Studies have shown that the tumor-free survival of patients after liver cancer conversion resection is related to the degree of pathological response, and the postoperative tumor-free survival is longer in patients with pathological response. In addition, tumor response is only based on imaging, not equivalent to pathological response, and there may be residual cancer cells. Therefore, when the transformed patients achieve the surgically resectable criteria, concurrent surgical treatment should be evaluated as soon as possible to clear necrotic tumor cells or viable tumor cells to achieve the pathological response criteria (52, 53). Timely surgery can also avoid tumor drug resistance and achieve better survival (54).

After the conversion is assessed to be successful, the timing of surgery should also be determined according to the preoperative conversion regimen. Expert consensus recommends: Before surgery, small-molecule targeted drugs (lenvatinib, apatinib, sorafenib, etc.) should be discontinued for more than 1~2 weeks; PD-1 inhibitors should be discontinued for more than 2-4 weeks, bevacizumab should be discontinued for >6 weeks, and bevacizumab should not be used until the wound fully recovers; if TACE or radiotherapy is performed, the surgery should be performed 4 weeks after the last treatment to reduce perioperative complications incidence and ensure the safety of surgery (5).

### Postoperative adjuvant therapy

There is still a lack of sufficient data and high-level evidence-based medical evidence to guide the selection of postoperative adjuvant therapy. However, the success of conversion implies that the tumor is sensitive to the regimen. Therefore, experts recommend that the original regimen or part of the drugs in the original regimen should be used for more than 6 months as appropriate according to the patient’s physical condition, adverse reactions and treatment tolerance. Re-examination should be performed every 3 months, and drug withdrawal can be considered when there is no tumor recurrence or metastasis in two consecutive imaging examinations, and tumor markers are normal for 3 consecutive months without upward trend (5).

### MDT is an important method to ensure the quality of conversion therapy

Due to the complex pathogenic factors, highly malignant biological behavior of liver cancer, great differences in liver disease backgrounds and prognosis, as well as different individual responses to treatment and the multiple disciplines involved (55), a multidisciplinary team (MDT) is required to evaluate the patients based on the imaging results to further provide individualized treatment regimen. During the treatment, the tumor response should be actively monitored, and the conversion regimen should be adjusted if necessary to create the opportunity for radical surgery with the ultimate goal to enable high-quality long-term survival for patients.

## Discussion

Molecule targeted drugs, represented by TKIs, have achieved promising therapeutic efficacy in existing clinical trials. Combined immunotherapy and local treatment may improve the ORR, increase the proportion of conversion resection rate, and prolong the survival time to benefit more patients with advanced HCC. In addition, PVE and ALPPS increase the residual liver volume, reduce the risk of postoperative liver failure, and ensure the safety of resection. Before choosing the treatment regimen, evaluating the cause of the unresectable, the patient’s liver function and performance status, and selecting an appropriate conversion method are necessary. The application of systemic treatment provides an opportunity for conversion and downstaging for patients with liver cancer at an intermediate and advanced stage and provides the possibility for surgical resection after conversion, thereby bringing hope to prolong overall survival and tumor-free survival.

At present, the research on systemic treatment is in the ascendant, but there are still many problems and challenges: (1) How to better screen the population with efficacy? (2) How to better arrange systemic treatment and local therapy to achieve downstaging effect? (3) How to choose a combination regimen to improve the conversion rate? (4) How to determine the conversion therapy time and arrange the operation time window? (5) Can ctDNA dynamic monitoring make up for the detection effect in patients with negative tumor indicators. More high-quality RCT studies are still needed to provide evidence-based medical data. In the future, higher-definition imaging technology, in conjunction with liquid biopsy, next-generation sequencing (NGS), and other techniques, could be used to assess the liver cancer tumor burden and metastasis in a more accurate and detailed manner in order to create a reasonable, individualized treatment plan for patients, thereby further improving the success rate of conversion and survival rate.

## Author contributions

HB, WM, and WC wrote the paper. ML and YY conceived the idea and supervised the manuscript. HB and WM contributed equally to this work. All authors contributed to the article and approved the submitted version.

## Funding

The study was partly supported by Natural Science Foundation of Guangdong Province (2018A030307076), Medical Science and Technology Funding of Guangdong Province (A2018369) and Clinical Research Project of Affiliated Hospital of Guangdong Medical University (LCYJ2021B002).

## Conflict of interest

The authors declare that the research was conducted in the absence of any commercial or financial relationships that could be construed as a potential conflict of interest.

## Publisher’s note

All claims expressed in this article are solely those of the authors and do not necessarily represent those of their affiliated organizations, or those of the publisher, the editors and the reviewers. Any product that may be evaluated in this article, or claim that may be made by its manufacturer, is not guaranteed or endorsed by the publisher.
